# Incidence and Risk Factors for Concurrent Syndromic Diagnosis in Presumed Idiopathic Developmental Dysplasia of the Hip

**DOI:** 10.5435/JAAOSGlobal-D-21-00169

**Published:** 2022-06-06

**Authors:** F. Keith Gettys, Adriana De La Rocha, Brandon A. Ramo

**Affiliations:** From the Shriners Hospitals for Children, Greenville, SC (Dr. Gettys), and the Department of Orthopedic Surgery, Texas Scottish Rite Hospital for Children, Dallas, TX (Dr. De La Rocha, and Dr. Ramo).

## Abstract

Infants referred for developmental dysplasia of the hip (DDH) may have a previously unidentified concomitant diagnosis of syndromic pathology. Our purpose was to examine the incidence of syndromic pathology in infants referred to a tertiary center with presumed idiopathic DDH and identify risk factors and difference in treatment courses between idiopathic and nonidiopathic cohorts.

**Methods:**

A retrospective analysis of a prospective cohort of infants younger than 3 years who were evaluated for DDH between 2008 and 2013 with a minimum 2-year follow-up. The clinical history and treatment were noted to determine the incidence and nature of concomitant syndromic diagnoses, after a confirmed diagnosis of DDH.

**Results:**

There were 202 patients: 177 were females (87.6%). Thirteen patients (6.4%) were later diagnosed with a neurologic/syndromic diagnosis. The workup leading to additional diagnosis was initiated by the orthopaedic surgeon in 8 of 13 patients (61.5%). Half of the referrals (4 of 8) made to other specialists were because of an abnormal treatment course (three—failure of typical DDH treatment and one—relapsed clubfeet). 7 of the 8 referrals were made because of developmental delays and decreased tone. 5 of the 13 nonidiopathic patients had other orthopaedic problems. The syndromic diagnoses included three cerebral palsy, two Kabuki syndrome, one Down syndrome, one myopathy, and one neuropathy. The diagnosis was made at an average of 2.3 years (0.04 to 4.7). No notable difference was observed in the incidence of the four known risk factors for DDH in syndromic patients compared with the idiopathic group. The syndromic patients required more open reductions (*P* = 0.002).

**Discussion:**

By the age of 3 years, 6% of the patients treated for DDH were found to have a syndrome or neurologic abnormality, and the referral for workup was made by the treating surgeon greater than 60% of the time.

The true etiology of developmental dysplasia of the hip (DDH) is unknown and is potentially multifactorial. Although several risk factors have been associated with DDH, the entire disease spectrum, ranging from ultrasonic acetabular changes or transient instability of the hip to fixed hip dislocations, can be seen in syndromic diagnoses, such as arthrogryposis, and in otherwise presumably normal, healthy children.^1^ Established risk factors are used to help screen and identify infants who may have the diagnosis and/or require treatment; however, their presence is not clearly related to whether the condition is idiopathic or associated with a syndrome.^2,3^ Infants referred for concerns of DDH may have a previously unidentified concomitant diagnosis of a syndrome, particularly because these infants are often referred early in life, before the opportunity to demonstrate other phenotypical characteristics associated with syndromes or medical diagnoses. Our purpose was to examine the incidence of syndromic pathology in infants referred to a tertiary center with presumed idiopathic DDH and identify risk factors and differences in treatment courses between idiopathic and nonidiopathic cohorts.

## Methods

This is an Institutional Review Board–approved review of a single-institution prospective database of patients referred for possible hip dysplasia between 2008 and 2013. Data collection consisted of demographic and clinical data, which included sex, birth history including intrauterine positioning, a family history of hip disease, treatment details, and any additional orthopaedic findings. Patients who initially presented with a known neuromuscular or syndromic diagnosis were excluded. A minimum 2-year follow-up was required. The final analysis was done on patients with confirmed diagnosis of DDH by the orthopaedic surgeon at the initial visit. DDH was defined by a hip that was dislocatable (Barlow-positive), reducible (Ortolani-positive), fixed, and dislocated and/or stable on examination but with evidence of dysplasia on ultrasonography.

The patient cohort was divided into two groups: (1) patients treated for DDH who were later identified with an underlying syndrome and considered to have nonidiopathic DDH and (2) patients with idiopathic DDH. The collected demographic and clinical data for the nonidiopathic patients were then compared with the idiopathic DDH patients.

Summary statistics were described with means and ranges for continuous variables and with frequencies for categorical variables. Differences between the nonidiopathic and idiopathic groups regarding baseline characteristics and treatment outcomes were analyzed using Student t-tests and Fisher exact tests with alpha *P* < 0.05. Statistical analysis was conducted using IBM SPSS Statistics 19 Software (IBM).

## Results

There were 202 patients: 25 male patients (12.4%) and 177 female patients (87.6%) were reviewed who were initially diagnosed with idiopathic DDH (55 [27%] bilateral/147 [73%] unilateral) (Table 1). Thirteen patients (6.4%), two males and 11 females, were later diagnosed with a neuromuscular or syndromic diagnosis and considered nonidiopathic DDH. The idiopathic group consisted of 189 patients (166 females and 23 males). No notable differences were observed in the incidence of the four known risk factors for DDH in syndromic patients compared with the idiopathic group. There were 11 female patients in the nonidiopathic group (84.6%) versus 166 (87.8%) in the idiopathic group, which was not significantly different (*P* = 0.666). None of the 13 syndromic patients had a family history of DDH compared with 26 (13.9%) in the idiopathic group, although this was not significantly different (*P* = 0.225). No difference was observed (*P* = 0.146) in the birth order rank between groups: 5 of 13 patients (38.5%) in the nonidiopathic group were born first compared with 99 of 166 patients (60.7%) in the idiopathic group. Four (30.8%) in the nonidiopathic group had a history of breech presentation compared with 61 (35.9%) in the idiopathic group (*P* = 1.00).

**Table 1:** Difference in Risk Factors for DDH Between Nonidiopathic and Idiopathic Groups

Factor	Nonidiopathic Group	Idiopathic Group	*P*
Female	11/13 (84.6%)	166/189 (87.8%)	0.666
Family history	0	26/189 (13.9%)	0.225
1st born	5/13 (38.5%)	99/189 (60.7%)	0.146
Breech	4/13 (30.8%)	61/189 (35.9)	1.000

**Patient**	**Sex**	**Age at Initial Examination**	**Syndromic Diagnosis (or Other Noted Conditions)**	**Age of Syndromic Diagnosis**	**Referred by Ortho**	**Age at the Date of Referral**	**Reason for Referral**	**Other Orthopaedic Conditions**	**Initial Examination Findings**	**Treatment**
1	F	2.27	Cerebral palsy	4 yrs 6 mo	Yes	2 yrs 3 mo	Short stature, dysmorphic features, and speech delays	Tight Achilles tendons	Fixed dislocation	OR/FNS/PO
2	F	1.66	Developmental delay and hypotonia	n/a	Yes	1 yr 8 mo	Delayed walking		Fixed dislocation	T/CR/PO
3	F	1.16	Developmental delays, strabismus, behavioral problems, and hearing issues	2 yrs 2 mo	Yes	2 yrs 2 mo	Delayed walking		Fixed dislocation	OR/FNS/PO
4	F	0.88	Kabuki syndrome	4 yrs 7 mo	Yes	1 yr 6 mo	Develop delays, abnormal postoperative course, and subluxation of R hip at 2nd cast change	Thumb duplication	Fixed dislocation	P/CR
5	F	0.70	Developmental delay and hypotonia	2 yrs 2 mo	Yes	1 yr 6 mo	Failed treatment and delayed milestones		Fixed dislocation	T/CR/OR/PO/FNS
6	M	0.37	Central core myopathy	10 mo	No	n/a	n/a		Fixed dislocation	P/B/OR
7	F	0.19	Kabuki syndrome	4 yrs 8 mo	Yes	1 yr 5 mo	Failed Pavlik	Left radius volar dislocation	Fixed dislocation	P/CR/OR/PO/FS
8	F	0.10	Cerebral palsy	1 yr 5 mo	No	n/a	n/a		Ultrasonic hip dysplasia	B/OR/PO/FNS
9	F	0.10	Chrom. 18 deletion, global developmental delay, G-tube, and microcephaly	2 yrs 8 mo	No	n/a	n/a		Ortolani-positive	P/OR
10	F	0.05	Developmental delay	1 yr 5 mo	No	n/a	n/a		Fixed dislocation	P/CR/OR/PO
11	M	0.04	Motor-sensory axonal polyneuropathy	1 yr 2 mo	Yes	8 mo	Relapse of clubfeet	Bilateral clubfeet	Fixed dislocation	OR/FNS
12	F	0.02	Down syndrome	1 mo	No	n/a	n/a		Right Ortolani-positive and left Barlow-positive	P/OR
13	F	0.02	Cerebral palsy	1 yr 9 mo	No	1 yr 10 mo	n/a	Toe walking	Barlow-positive	P

CR = underwent closed reduction, FNS = underwent femoral shortening, OR = underwent open reduction, P = treatment with Pavlik harness, PO = underwent a pelvic osteotomy

The syndromic diagnoses included three patients with a form of cerebral palsy, two patients with Kabuki syndrome, one patient with Down syndrome, one patient with central core myopathy, and one patient with motor-sensory polyneuropathy. The diagnosis was made at an average of 2.3 years (range 0.04 to 4.7). The additional five patients were thought to be syndromic and labeled as such by the treating physician. Seven were referred for genetic and/or neurologic evaluation, and three did not have a formal diagnosis documented at the most recent follow-up.

The workup leading to additional diagnosis was initiated by the treating orthopaedic surgeon in 8 of 13 of these patients (61.5%). Half of the referrals (4 of 8) made to other specialists were because of an abnormal or failed treatment course. Three patients had failure of established treatment for DDH: Two failed Pavlik harness treatment and one had late hip subluxation, leading the provider to consider an underlying syndrome. Two of these patients failing established treatment were diagnosed with Kabuki syndrome. One patient had early relapse of a clubfoot leading to referral. This patient was diagnosed with a motor-sensory polyneuropathy. Seven of the eight referrals were made because of developmental delays and decreased tone. One patient with dysmorphic facial features and speech delay was diagnosed with cerebral palsy.

Five of the 13 nonidiopathic patients had other orthopaedic problems. One patient with a tight Achilles tendon and one patient who was toe walking were both later diagnosed with cerebral palsy. A patient with bilateral clubfoot was diagnosed with a motor-sensory polyneuropathy. Two patients had upper extremity involvement, a thumb duplication, and a volar wrist dislocation and were diagnosed with Kabuki syndrome.

The mean age of presentation for the nonidiopathic group was 0.58 years (range 0.02 to 2.27) compared with the idiopathic mean age of 0.50 years (range 0.01 to 2.88). The difference between the two groups was found not to be notable. The nonidiopathic group's initial hip examination revealed that six patients (46.2%) had bilateral involvement compared with 48 idiopathic patients (25.4%) with bilateral involvement in the idiopathic group (*P* = 0.113). Infants in the nonidiopathic group had a significantly greater number of fixed dislocations than patients in the idiopathic group (69.2% versus 37.6%, *P* = 0.037).

Pavlik harness was used for treatment in 6 of the nonidiopathic patients (46.2%) compared with 124 of the idiopathic patients (65.6%) (*P* = 0.229). The differences in ages of treatment were not statistically different (*P* = 0.303). Of those six patients treated initially with a Pavlik harness, all but one (diagnosed with cerebral palsy) had required additional treatment at the time of the latest follow-up. Closed reduction was done in 4 nonidiopathic (30.8%) and 45 idiopathic (23.8%) patients. Three of the four patients in the nonidiopathic group have gone on to require more surgery indicating a success of only 25%. By contrast, in the idiopathic group, only 12 patients (26.7%) required additional surgery (closed reduction or pelvic osteotomy) after closed reduction reflecting a difference from the nonidiopathic group (n = 3, 75%, *P* = 0.079). Nine nonidiopathic patients (69.2%) primarily (6) or eventually (3 after previous closed reduction) underwent open reduction compared with 48 of the 189 idiopathic patients (25.4%), which was a significant difference (*P* = 0.002). Six patients of the nonidiopathic group (46.2%) at the past follow-up had undergone a pelvic osteotomy compared with only 9 of the 189 patients in the idiopathic group (4.8%) (*P* < 0.001).

## Discussion

Infantile DDH presents in many forms requiring different methods and durations of treatment. Some of the factors that affect the treatment and the overall outcome have been studied.^3,4^ Like many other orthopaedic diagnoses such as clubfoot and scoliosis, there are forms of DDH that are idiopathic and those that occur in association with a syndrome. These nonidiopathic forms frequently have different natural histories and do not always respond to the same methods of treatment.^5,6^ Similarly, hip dysplasia associated with neuromuscular disorders is different from idiopathic DDH. One purpose of our study was to identify whether there were any factors that could alert the treating physician to a possible nonidiopathic form of DDH when examining a presumably normal infant with DDH. In addition, we sought to understand whether the presentation of idiopathic-appearing DDH later found associated with a syndromic diagnosis had any difference regarding treatment outcomes.

The known risk factors of female sex, first born, breech birth history, and family history are well known and have been studied.^3^ In our study, we analyzed whether there were differences in these rates in idiopathic compared with nonidiopathic DDH cohorts. We found no differences in the rates of these classic risk factors between our cohorts indicating that they may not be a useful indicator of the presence of other disease pathology to the treating physician. Obviously, the presence of another orthopaedic anomaly may be an indicator of an underlying syndrome, but in our series, 7 of 13 had no other orthopaedic manifestations.

The spectrum of DDH is quite diverse, and thus, the treatment results are quite variable. In our study, the rate of use of the Pavlik harness was not markedly different in either group but nearly universally failed in the nonidiopathic group because all but one of the patients had undergone additional treatment after the harness. Similarly, there was no difference in the rates of patients undergoing closed treatment between the two groups; however, there was a difference in those requiring additional surgery after closed reduction attempts in the nonidiopathic group (75% versus 24.4%). Furthermore, we found that there were markedly more open reductions and pelvic osteotomies required in the nonidiopathic group relative to the idiopathic DDH patients. In general, our conclusion is that syndromic DDH is more likely to fail with established treatment methods, and therefore, the failure of these modalities should raise suspicion for a nonidiopathic diagnosis to the treating physician. It seems not surprisingly that these are different entities with different natural histories despite similar initial presentations.

Infantile DDH has been studied along with other musculoskeletal diagnoses looking for associations between various diagnoses.^1,7,8^ In our study, several other orthopaedic physical findings were documented in some of the patients with nonidiopathic DDH. The presence of toe walking and a tight Achilles tendon was observed in two of the nonidiopathic infants who were later diagnosed with cerebral palsy. Owing to the nature and size of our relatively small population of nonidiopathic patients, an association with other findings cannot be fully made. However, in the two patients with upper extremity findings, both of them were diagnosed with Kabuki syndrome. Kabuki syndrome is a genetic disorder that is named for the classical makeup worn in Kabuki theater.^9^ The disorder is characterized by facial features, various cognitive disabilities, and various skeletal anomalies. There have been several articles discussing the hip involvement associated with this syndrome with an incidence of 12%.^9,10^ These patients were also noted to have decreased tone and ligamentous laxity contributing to a more complex treatment course. Both patients in our study had attempts at Pavlik harness but failed and went on to closed reductions. One of them required additional surgery, including an open reduction, femoral shortening, and pelvic osteotomy at the last follow-up.

One other patient was referred for a neurologic evaluation after failed clubfoot treatment. This patient was ultimately diagnosed with motor-sensory polyneuropathy, type unspecified. Delayed milestones, dysmorphic facial features, and decreased tone were other reasons documented for referral for a syndromic workup in our patients.

The examination of a newborn or a young infant is often challenging. In addition, it is easy to become focused on the treatment of the identified problem such as hip dislocation and fail to repetitively fully assess the developing child attached to the hip. Our study demonstrated that failure of established methods of age-appropriate treatment such as the Pavlik harness or closed reduction seemed to be a clue to identifying a nonidiopathic diagnosis. In addition, the treating physician must remain aware of other pertinent, sometimes subtle physical examination features such as dysmorphic facial features or upper extremity anomalies. In recognizing subtle difference and the absence of a normal treatment course, eight nonidiopathic patients (60%) were referred for and identified to have a neuromuscular or syndromic diagnosis by the orthopaedic surgeon. We were not able in this retrospective review to specifically assess how accurate the treating surgeon was at identifying syndromic pathology, i.e., how many patients received a second diagnosis versus how many total patients were referred and deemed otherwise normal.

This study has several potential limitations. First, there is sample bias because our groups came from patients referred to a tertiary institution for the treatment of DDH. By doing so, the assumption is made that these patients represent all patients with infantile DDH.

Second, although the patients were collected prospectively as part of a DDH study, a chart review to identify secondary pathology was conducted in a retrospective nature and is therefore subject to the limitations of accuracy and completeness of documentation of all retrospective studies. For example, there might have been other potential physical or development findings or clues to diagnosis that could have helped lead to the diagnosis that was not well documented.

Third, our study only took place at one center over a 5-year period leading to a small number of syndromic patients, making comparisons with the idiopathic cohort somewhat limited in their statistical power. Our minimum follow-up was 2 years, and with longer follow-up, additional syndromic diagnoses and other risk factors might be elucidated. Despite this, the 6% incidence we have documented of nonidiopathic pathology reflects a large enough percentage of the whole to heed awareness by treating surgeons.

In conclusion, by the age of 3 years, 6% of the patients treated for DDH were found to have a syndrome or neurologic abnormality, which increases the likelihood of open treatment. The referral for workup was made by the treating surgeon greater than 60% of the time, indicating an important role for the orthopaedic surgeon in the screening for other diagnoses. The diagnosis was made at an average age of 2.3 years, and the most common finding in these patients was global developmental delay. Providers should be mindful of the potential presence of other pathology in “idiopathic DDH.”

## References

[1] Loder RT Skopelja EN: The epidemiology and demographics of hip dysplasia. ISRN Orthop 2011;2011:238607. 24977057 10.5402/2011/238607 PMC4063216

[2] Dunn PM: Perinatal observations on the etiology of congenital dislocation of the hip. Clin Orthop Relat Res 1976:11-22. 954299

[3] Weinstein SL Mubarak SJ Wenger DR: Developmental hip dysplasia and dislocation: Part I. Instr Course Lect 2004;53:523-530. 15116641

[4] Weinstein SL Mubarak SJ Wenger DR: Developmental hip dysplasia and dislocation: Part II. Instr Course Lect 2004;53:531-542. 15116642

[5] Janicki JA Narayanan UG Harvey B Roy A Ramseier LE Wright JG: Treatment of neuromuscular and syndrome-associated (nonidiopathic) clubfeet using the Ponseti method. J Pediatr Orthop 2009;29:393-397. 19461383 10.1097/BPO.0b013e3181a6bf77

[6] Moroney PJ Noël J Fogarty EE Kelly PM: A single-center prospective evaluation of the Ponseti method in nonidiopathic congenital talipes equinovarus. J Pediatr Orthop 2012;32:636-640. 22892629 10.1097/BPO.0b013e31825fa7df

[7] Paton RW Choudry QA Jugdey R Hughes S: Is congenital talipes equinovarus a risk factor for pathological dysplasia of the hip? A 21-year prospective, longitudinal observational study. Bone Joint J 2014;96-B:1553-1555. 25371473 10.1302/0301-620X.96B11.34130

[8] Minihane KP Grayhack JJ Simmons TD Seshadri R Wysocki RW Sarwark JF: Developmental dysplasia of the hip in infants with congenital muscular torticollis. Am J Orthop (Belle Mead NJ) 2008;37:E155-E158. 18982188

[9] Ramachandran M Kay RM Skaggs DL: Treatment of hip dislocation in Kabuki syndrome: A report of three hips in two patients. J Pediatr Orthop 2007;27:37-40. 17195795 10.1097/BPO.0b013e31802b70cf

[10] Wada A Nakamura T Yamaguchi T : Surgical treatment of hip dislocation in Kabuki syndrome: Use of incomplete periacetabular osteotomy for posterior acetabular wall deficiency. J Child Orthop 2012;6:261-267. 23904891 10.1007/s11832-012-0426-y PMC3425692

